# Evaluation of cebranopadol, a dually acting nociceptin/orphanin FQ and opioid receptor agonist in mouse models of acute, tonic, and chemotherapy-induced neuropathic pain

**DOI:** 10.1007/s10787-017-0405-5

**Published:** 2017-10-25

**Authors:** Kinga Sałat, Anna Furgała, Robert Sałat

**Affiliations:** 1 Chair of Pharmacodynamics, Department of Pharmacodynamics, Jagiellonian University Medical College, 9 Medyczna St, 30-688 Krakow, Poland; 2 Faculty of Production Engineering, Warsaw University of Life Sciences, 164 Nowoursynowska St, 02-787 Warsaw, Poland

**Keywords:** Cebranopadol, Pain models, Neurogenic inflammation, Chemotherapy-induced peripheral neuropathy, Oxaliplatin, Simvastatin

## Abstract

**Background:**

Cebranopadol (a.k.a. GRT-6005) is a dually acting nociceptin/orphanin FQ and opioid receptor agonist that has been recently developed in Phase 2 clinical trials for painful diabetic neuropathy or cancer pain. It also showed analgesic properties in various rat models of pain and had a better safety profile as compared to equi-analgesic doses of morphine. Since antinociceptive properties of cebranopadol have been studied mainly in rat models, in the present study, we assessed analgesic activity of subcutaneous cebranopadol (10 mg/kg) in various mouse pain models.

**Methods:**

We used models of acute, tonic, and chronic pain induced by thermal and chemical stimuli, with a particular emphasis on pharmacoresistant chronic neuropathic pain evoked by oxaliplatin in which cebranopadol was used alone or in combination with simvastatin.

**Key results:**

As shown in the hot plate test, the analgesic activity of cebranopadol developed more slowly as compared to morphine (90–120 min vs. 60 min). Cebranopadol displayed a significant antinociceptive activity in acute pain models, i.e., the hot plate, writhing, and capsaicin tests. It attenuated nocifensive responses in both phases of the formalin test and reduced cold allodynia in oxaliplatin-induced neuropathic pain model. Its efficacy was similar to that of morphine. Used in combination and administered simultaneously, 4 or 6 h after simvastatin, cebranopadol did not potentiate antiallodynic activity of this cholesterol-lowering drug. Cebranopadol did not induce any motor deficits in the rotarod test.

**Conclusion:**

Cebranopadol may have significant potential for the treatment of various pain types, including inflammatory and chemotherapy-induced neuropathic pain.

## Introduction

The International Association for the Study of Pain (IASP) defines pain as an unpleasant sensory and emotional experience associated with actual or potential tissue damage, or described in terms of such damage. The sensation of pain involves multiple signaling pathways, numerous neurotransmitters, and other mediators that are involved in the inhibitory or facilitatory control of pain intensity. These mechanisms affect the perception of stimuli as non-painful or painful, respectively, but their positive or negative modulation of pain signaling is strongly dependent on the receptor type involved and its location in the target tissue (Argoff 2011).

In living organisms, endogenous opioids (endorphins, enkephalins, and dynorphins) are key molecules in the descending pain suppression pathways. Recently, it has been discovered that opioid receptors are widely distributed not only in the central but also peripheral nervous system and in the non-neuronal tissues. There is also evidence from animal and human studies for the involvement of peripheral opioid receptors in analgesia, especially in the presence of inflammation (Sehgal et al. 2011), or neuropathy (Plein and Rittner 2017).

The nociceptin/orphanin FQ opioid peptide receptor (NOP receptor) is the most recently discovered member of the opioid receptor family. Together with its endogenous ligand–nociceptin, also known as orphanin FQ (N/OFQ), it forms the fourth member of the opioid receptor family which is abundantly expressed in various body tissues. A large body of evidence shows that the activation of N/OFQ-NOP system regulates functions of the central nervous system being implicated in feeding, body weight homeostasis, stress, stress-related psychiatric disorders—depression, anxiety, drug, and alcohol dependence (Witkin et al. 2014). Data from preclinical studies are also in line with these findings showing that N/OFQ plays an important role in comorbid neuropathic pain and post-traumatic stress disorder (Zhang et al. 2015), acute and chronic restraint stress responses (Delaney et al. 2012), depression (Vitale et al. 2017), and other stress-related conditions (Leggett et al. 2006; Witkin et al. 2014).

Apart from this, the N/OFQ-NOP pathway is also involved in the modulation of inflammatory and immune responses of the body by influencing migration of leucocytes, cytokine secretion, and lymphocyte proliferation. Recent findings showing the involvement of N/OFQ in inflammatory responses (Gavioli and Romão 2011) and the evidence for a role of NOP receptors and N/OFQ in the modulation of neurogenic inflammation, migraine (Tajti et al. 2015), and airway tone (Singh et al. 2016) led to the hypothesis that N/OFQ-NOP system might be an important drug target for analgesic drugs. This is in part supported by the previous findings showing that the blockade of NOP receptors can attenuate inflammation (Gavioli et al. 2015). On the other hand, this issue is not completely clear and unequivocally explored as there is also evidence for analgesic efficacy of NOP agonists in neuropathic and inflammatory pain, both in animal models (Sukhtankar et al. 2013) and clinical trials (Sałat et al. 2015a).

Cebranopadol (a.k.a. GRT-6005; trans-6′-fluoro-4′,9′-dihydro-N,N-dimethyl-4-phenyl-spiro[cyclohexane-1,1′(3′H)-pyrano[3,4-b]indol]-4-amine) is a dually acting nociceptin/orphanin FQ and opioid receptor agonist (K_i_ (nM)/EC_50_ (nM)/relative efficacy (%): human NOP receptor 0.9/13.0/89; human mu-opioid peptide (MOP) receptor 0.7/1.2/104; human kappa-opioid peptide (KOP) receptor 2.6/17/67; human delta-opioid peptide (DOP) receptor 18/110/105) (Linz et al. 2014) that has been recently developed in Phase 2 clinical trials for painful diabetic neuropathy or cancer pain (reviewed in Sałat et al. 2015a). It showed analgesic properties in various rat models of acute thermal pain, i.e., tail-flick model, chronic inflammatory pain (CFA-induced arthritis model), bone cancer pain model (Raffa et al. 2017), and neuropathic pain: chronic constriction injury (CCI) and diabetic neuropathic pain models (Raffa et al. 2017) after intraplantar, intracerebroventricular, intrathecal, intravenous (Tzschentke et al. 2017), subcutaneous, or oral route (Linz et al. 2014). Compared to selective MOP receptor agonists, cebranopadol was more potent in models of chronic neuropathic than acute nociceptive pain and its duration of action was long (Linz et al. 2014). Noteworthy, safety pharmacology studies with cebranopadol demonstrated that the development of analgesic tolerance in cebranopadol-treated animals subjected to CCI procedure was delayed as compared to equi-analgesic doses of morphine (Sałat et al. 2015a), and at analgesic doses, cebranopadol did not cause respiratory depression in a rat whole-body plethysmography model, or motor coordination deficits in the rat rotarod test (Linz et al. 2014, 2017; Lambert et al. 2015; Günther et al. 2017).

The data presented above come from rat studies and there is limited knowledge about antinociceptive properties of cebranopadol in mice. Moreover, these previous studies investigated the influence of cebranopadol on tactile allodynia but not thermal (i.e., heat or cold) allodynia and hyperalgesia. Hence, in the present study, we utilized mouse models of acute, tonic, and chronic pain induced by thermal or chemical (inflammatory) stimuli, with a particular emphasis on pharmacoresistant chronic neuropathic pain evoked by oxaliplatin. Oxaliplatin is a third-generation platinum-based anti-tumor drug used to treat advanced colorectal cancer. Compared to other platinum-based drugs, it has lower incidence of hematological adverse effects and gastrointestinal toxicity, but in approximately 95% of patients, oxaliplatin causes severe neuropathic pain episodes and increased sensitivity to cold (Manji 2013) which often lead to dose reduction or even treatment discontinuation. These neuropathic pain episodes can be effectively attenuated by μ opioid peptide (MOP) and NOP receptor agonists (Micheli et al. 2015).

In the present study, we investigated the effect of cebranopadol on cold nociceptive threshold of oxaliplatin-treated mice. We used two protocols of its administration: the first one which utilized this drug alone, and the second one in which cebranopadol was used in combination with simvastatin. Available data show potential effectiveness of simvastatin in several animal models of pain (Shi et al. 2011; Miranda et al. 2011; Chen et al. 2013; Mansouri et al. 2017), including inflammatory (Chen et al. 2013) and neuropathic pain models (Shi et al. 2011). First, in the previous studies (Bhalla et al. 2015), simvastatin effectively reversed vincristine-induced neuropathic pain by anti-inflammatory effects and it was able to attenuate vincristine-induced increase in myeloperoxidase activity. Second, anti-inflammatory and anti-oxidant effects of this drug also resulted in reduction of cisplatin-induced nephrotoxicity and hepatotoxicity in rats (Işeri et al. 2007) and simvastatin protected Sertoli cells against cisplatin cytotoxicity (Wang et al. 2015). Third, it has been also shown that simvastatin was able to attenuate neuropathic pain induced by CCI in rats and it significantly decreased the ratio of membrane/cytosolic RhoA by reducing RhoA/LIMK/cofilin pathway activity (Qiu et al. 2016). Furthermore, it exerted antihyperalgesic and antiallodynic effects through the inhibition of spinal RhoA activation and its daily intrathecal administration before nerve injury prevented the development of neuropathy in nerve-ligated mice (Ohsawa et al. 2016). Interestingly, the RhoA-dependent pathway is implicated in the regulation of Transient receptor potential melastatin subtype 8 (TRPM8), a cold-sensing cation channel (Sun et al. 2014) which is also required for cold-related symptoms of oxaliplatin-induced peripheral neurotoxicity (Knowlton et al. 2011; Kono et al. 2012). Taken together, these data clearly suggest that statins are effective in neuropathic pain conditions and they might modulate pain sensitivity of cold-exposed subjects. This justifies the rationale to undertake this part of research which aimed to assess if combined use of simvastatin and cebranopadol could attenuate cold hypersensitivity of oxaliplatin-treated mice.

## Materials and methods

### Animals and housing conditions

Experiments were carried out at the Department of Pharmacodynamics, Faculty of Pharmacy, Jagiellonian University Medical College in Krakow. The investigators involved in behavioral assays were blinded to the experimental groups to avoid potential bias in data recording. Adult male Albino Swiss (CD-1) mice weighing 18–22 g were purchased from the Animal Breeding Farm of the Jagiellonian University Faculty of Pharmacy. Before behavioral tests, the animals were kept in groups of 10 mice in standard plastic cages and housed under controlled conditions (room temperature of 22 ± 2 °C, light/dark (12:12) cycle, lights on at 8.00 AM, humidity 50–60% and free access to food and water). Experimental groups consisted of 8–10 animals/dose. For the tests, the animals were selected randomly. After the assay, the mice were immediately euthanized by cervical dislocation. All experiments were performed between 9 AM and 3 PM. The procedures for in vivo tests were approved by the Local Ethics Committee of the Jagiellonian University in Krakow (Approval No. 4/2016; 22.03.2016) and the treatment of animals was in full accordance with ethical standards laid down in respective Polish and EU regulations (Directive No. 86/609/EEC).

### Chemicals

Cebranopadol and morphine hydrochloride were purchased from MedChem Express (NJ, USA) and Polfa Kutno (Poland), respectively. These drugs at a fixed dose of 10 mg/kg were administered subcutaneously before behavioral tests. This dose was selected based on our previous pilot study which revealed that full antinociceptive efficacy of morphine used as a reference drug was observed at doses 6–10 mg/kg. For in vivo tests, cebranopadol was prepared in a mixture of 100% DMSO (Polskie Odczynniki Chemiczne, Poland) and 0.9% saline (1:1), then being diluted in saline to achieve a proper concentration and was injected 120 min before testing (for details, see Sect. 3.1.1). Morphine hydrochloride was dissolved in 0.9% saline solution and it was administered 60 min before the tests. Control animals received vehicle. Acetic acid, ethanol, 0.9% natrium chloride solution, 5% glucose solution, and 37% formaldehyde solution were provided by Polskie Odczynniki Chemiczne (Poland). Capsaicin, simvastatin, and oxaliplatin were purchased from Sigma-Aldrich (Germany). For the in vivo experiments, capsaicin was dissolved in ethanol (100%) at 5% of the final desired volume, and then, 0.9% saline was added. This mixture was vortexed for 10 min (Sałat et al. 2014). Simvastatin was suspended in 0.9% saline solution. The dose of simvastatin used in the present research (100 mg/kg, p.o.) was chosen on the basis of the previous studies published by other authors (Mansouri et al. 2015).

### Behavioral tests

#### Acute pain models (thermal pain, inflammatory, and chemogenic pain models)

##### Thermal pain—hot plate test

Antinociceptive properties of cebranopadol and morphine in the hot plate test were assessed as described previously (Eddy and Leimbach 1953) with some minor modification (Sałat et al. 2015b). Briefly, 1 day before the proper pharmacological experiment, the animals were tested to establish baseline pain sensitivity threshold (baseline latency) for each animal. For further pain tests, only mice with baseline latencies ≤ 20 s were used. On the test day, the animals were subcutaneously treated either with the test drugs, or vehicle 60 min before placing the animal on a hot/cold plate apparatus (Bioseb, France). This apparatus can generate heat or cold and is supplied with a temperature controller that maintains surface temperature to a set point. Herein, the temperature was set at 55–56 °C. Latency time to pain reaction, i.e., the time until the animal licked its hind paws or jumped was recorded by means of a stop-watch (Q&Q HS-46, Japan, precision: 1/100 s). In this assay, a cut-off time (60 s) was enforced to avoid paw tissue damage, and mice not responding within 60 s were removed from the apparatus and assigned a score of 60 s.

##### Inflammatory acute pain—writhing test

In this test, mice were placed individually into glass beakers and were allowed to habituate for the next 30 min. Then, each mouse was weighed, injected with the test compound or vehicle, and then placed back into the glass beaker for 60 min. To induce inflammatory pain, 0.9% acetic acid solution prepared in saline was injected by the intraperitoneal route. Mice were placed in the beakers once again and were observed continuously for the next 30 min. Stereotypical writhes (lengthwise constrictions of the torso with a concomitant concave arching of the back) were counted over this period in drug-treated and control mice (Sałat et al. 2013a).

##### Neurogenic pain—capsaicin test

After the adaptation period (15 min), 1.6 μg of capsaicin dissolved in 20 μl of a mixture containing 0.9% saline and ethanol (5% of the final volume) was injected intraplantarly in the ventral surface of the right-hind paw of each mouse. The animals were observed individually for 5 min following capsaicin injection. In all experimental groups, the amount of time spent on licking, biting, or lifting the injected paw was recorded with a chronometer and was considered as an indicator of nociception (Sałat et al. 2014).

#### Inflammatory tonic pain model—formalin test

In rodents, the injection of diluted formalin solution evokes a biphasic nocifensive behavioral response (licking or biting the injected paw) of experimental animals. The first (acute) nociceptive phase of the test lasts for 5 min, whilst the second (late) one occurs between 15 and 30 min after formalin injection. In this assay, 20 μl of 5% formalin solution was injected into the dorsal surface of the right-hind paw of each mouse. Then, the mice were placed separately in glass beakers and were observed for the next 30 min. Total time spent on licking or biting the formalin-treated paw was measured during the first 5 min of the test, and then between 15 and 30 min of the test in drug-treated and vehicle-treated mice (Sałat et al. 2015b).

#### Chemotherapy-induced neuropathic pain model

##### Induction phase

To develop chemotherapy-induced peripheral neuropathy (CIPN) and neuropathic pain mice were injected intraperitoneally with a single dose of oxaliplatin (10 mg/kg prepared in 5% glucose solution). Control mice received 5% glucose solution as vehicle. Pain threshold of experimental animals was assessed using the cold plate test 3 h and 7 days after oxaliplatin administration to establish its effect on acute-phase and late-phase cold allodynia, respectively.

##### Influence on cold allodynia (cold plate test)—single-drug administration protocol

**Fig. 1:**
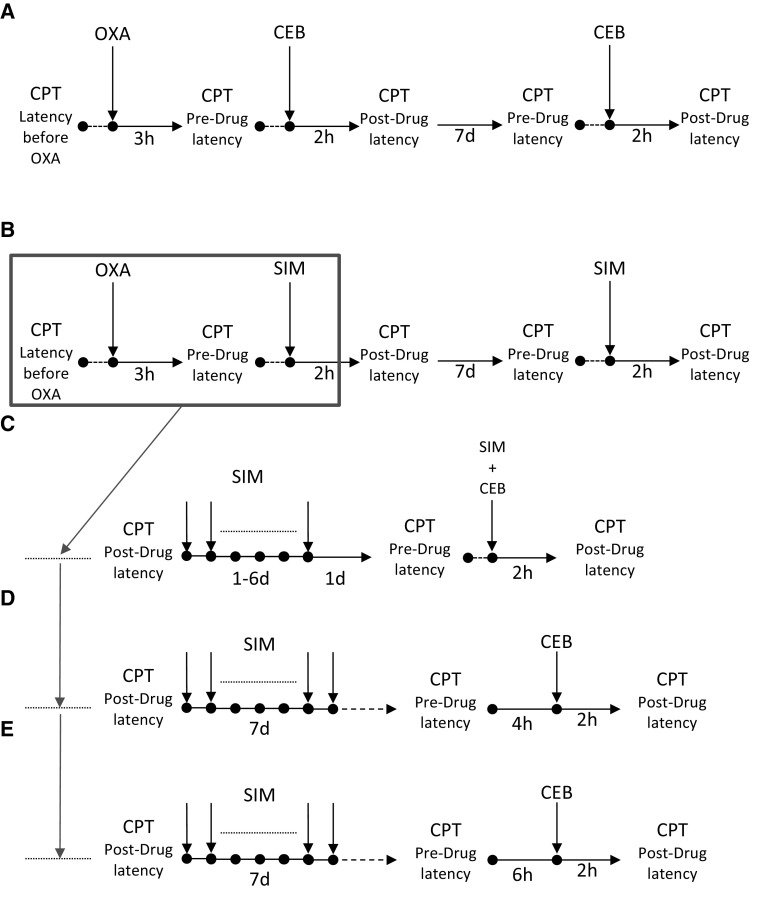
Protocol of the administration of cebranopadol alone (**a**), simvastatin alone (**b**), or both drugs in combination (**c**–**e**) in oxaliplatin-induced neuropathic pain model. The scheme shows specific time-points at which the data measured as latencies to pain reaction in the cold plate test were collected. *CPT* cold plate test, *OXA* oxaliplatin, *CEB* cebranopadol, *SIM* simvastatin

The cold plate test was performed using the hot/cold plate apparatus set at 2 °C. In this assay (Fig. 1a), the animals were tested first to obtain baseline latencies to pain reaction (i.e., lifting, biting, shaking of hind paws, jumping, and movement deficits) before oxaliplatin injection (referred to as latencies ‘before oxaliplatin’). Then, oxaliplatin was injected and latencies to pain reaction were measured again (referred to as ‘pre-drug latencies’). Subsequently, test drugs were administered and post-drug latencies were measured again. In this assay, a cut-off time of 60 s was established to avoid potential paw tissue damage and animals not responding within 60 s were removed from the apparatus and assigned a score of 60 s.

##### Influence on cold allodynia (cold plate test)—combined drug administration protocol (simvastatin and cebranopadol)

In the cold plate test, we additionally investigated the antiallodynic activity of cebranopadol used in combination with simvastatin. The protocol of the administration of these both drugs (alone or in combination) is shown in Fig. 1. It served to establish whether cebranopadol could enhance antiallodynic properties of simvastatin in the oxaliplatin model of neuropathic pain.

#### Influence on motor coordination—rotarod test

Before the test, the animals were trained for 3 consecutive days on the rotarod apparatus (Rotarod apparatus, May Commat RR0711, Turkey; rod diameter: 2 cm) that was rotating at a fixed speed of 18 rotations per minute (rpm). In each session, the mice were placed on the rotating rod for 3 min with an unlimited number of trials. The proper experiment was performed 24 h after the last training session. After the administration of the test drugs or vehicle, the mice were tested on the rod that revolved at 6, 18, or 24 rpm. Motor impairments in mice were defined as the inability to remain on the rotarod apparatus for 1 min. The results are expressed as the mean time spent on the rotarod (Sałat et al. 2015b).

### Data analysis

Data analysis was carried out using GraphPad Prism software (v.5.0, CA, USA). Numerical results are expressed as the mean ± SEM. Statistical analysis was carried out using one-way analysis of variance (ANOVA), followed by Tukey’s, or Dunnett’s post hoc comparisons, or repeated-measures analysis of variance, followed by Bonferroni’s multiple comparison. *P* < 0.05 was considered significant.

## Results

### Acute pain models

#### Thermal acute pain—hot plate test

**Fig. 2:**
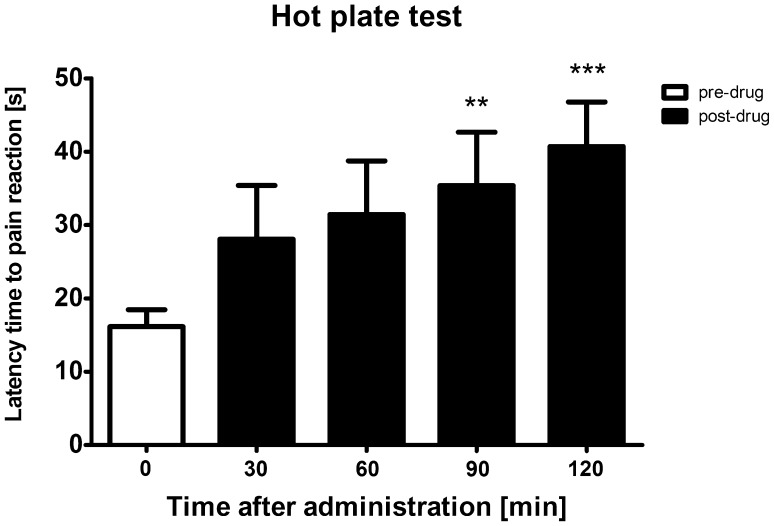
Antinociceptive activity of subcutaneous cebranopadol in the mouse hot plate test measured at various time-points. Results are shown as latency time to pain reaction (±SEM) in response to thermal stimulation (temperature of 55–56 °C). Statistical analysis: repeated-measures analysis of variance, followed by Bonferroni’s multiple comparison. Significance vs. pre-drug latency: **p < 0.01, ***p < 0.001

To establish at which time-points subcutaneous cebranopadol exerts antinociceptive activity, the mouse hot plate test was used. In this assay, an overall effect of treatment was observed (*F*[4,28] = 6.676, *p* < 0.001). The post hoc analysis revealed that the antinociceptive activity of cebranopadol was significant 90 min and 120 min after its administration (*p* < 0.01 and *p* < 0.001, respectively; Fig. 2). Taking this into consideration, further pain tests were performed 90 and 120 min after cebranopadol injection.

Previously obtained results for subcutaneous morphine in the hot plate test performed under the same experimental conditions (Sałat et al. 2009) demonstrated its significant antinociceptive properties. The dose of 6 mg/kg was highly effective and this activity was noted earlier than that of cebranopadol (between 30 and 60 min after administration).

#### Inflammatory acute pain—writhing test

**Fig. 3:**
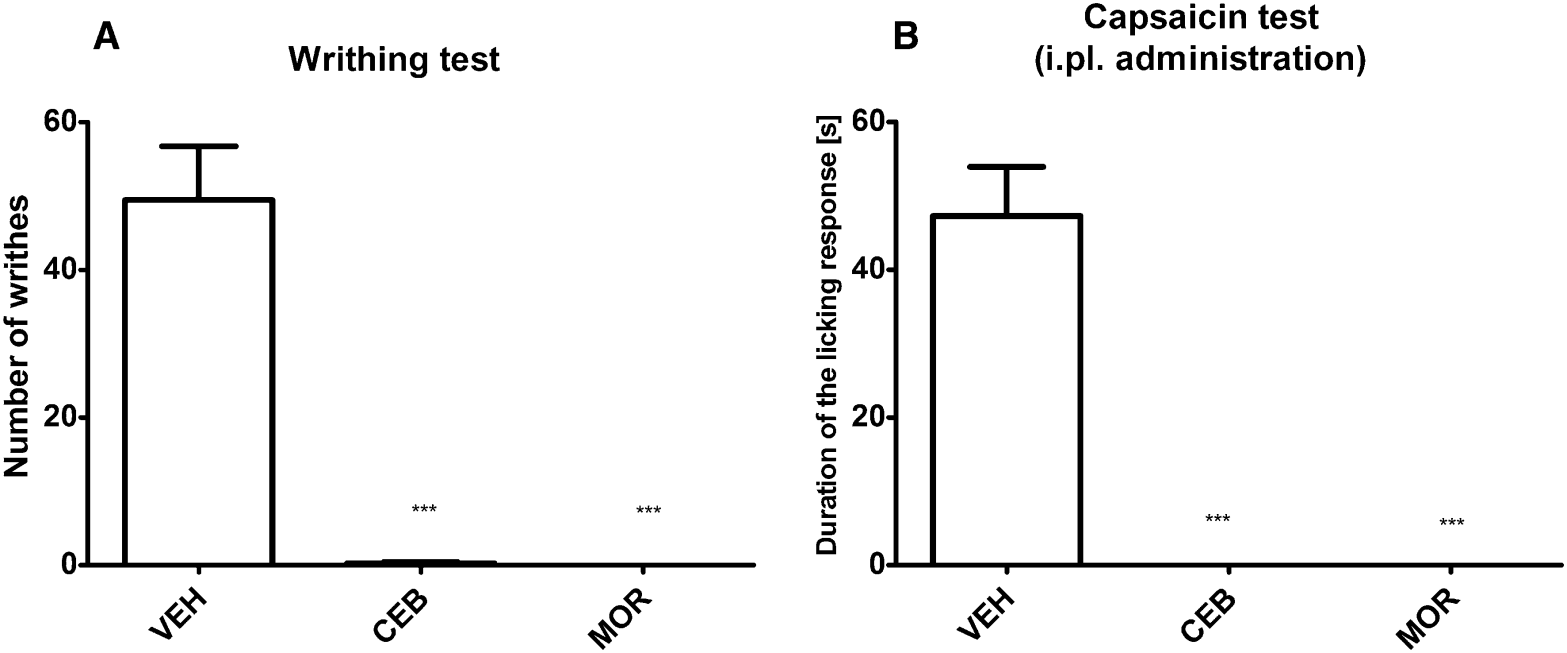
Antinociceptive activity of cebranopadol (CEB) and morphine (MOR) in the acetic acid-induced writhing test (**a**) and in capsaicin-induced neurogenic pain model (**b**). Results are shown as the mean number of writhes (±SEM) (**a**), or duration of the licking/biting response (±SEM) (**b**). Statistical analysis: one-way analysis of variance, followed by Dunnett’s post hoc test. Significance vs. vehicle-treated mice: ***p < 0.001

As shown in Fig. 3a, cebranopadol and morphine were equally effective in reducing acetic acid-induced writhing behavior (*F*[2,20] = 52.86, *p* < 0.0001).

#### Neurogenic pain—capsaicin test

In the intraplantar capsaicin test (Fig. 3b), the pretreatment with cebranopadol and morphine completely abolished the licking response of capsaicin-treated mice (*F*[2,21] = 50.18, *p* < 0.0001). This confirmed strong antinociceptive properties of both agents in this pain model.

### Inflammatory tonic pain model—formalin test

**Fig. 4:**
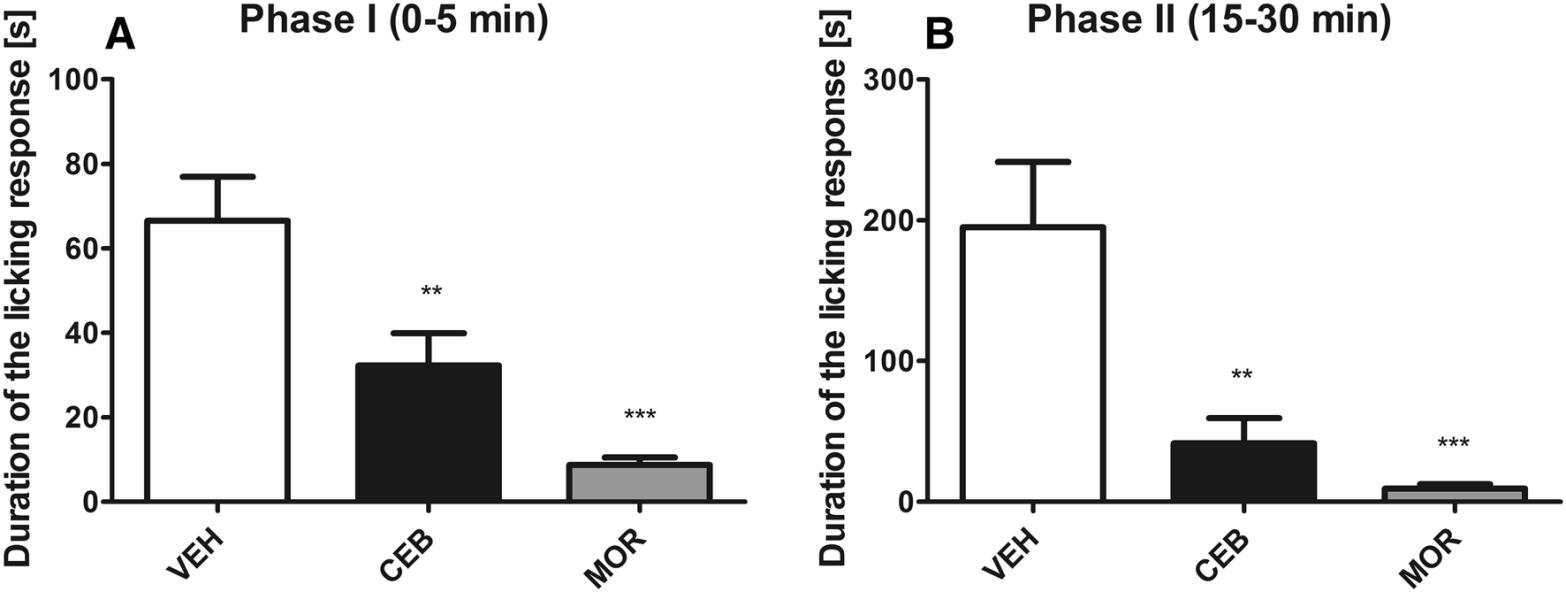
Antinociceptive activity of cebranopadol (CEB) and morphine (MOR) in the tonic pain model (i.e., the intraplantar formalin test). Influence on the first, neurogenic phase responses (**a**) and on the second, inflammatory phase responses (**b**). Statistical analysis: one-way analysis of variance, followed by Dunnett’s post hoc test. Significance vs. vehicle-treated mice: **p < 0.01, ***p < 0.001

The results obtained in the first phase of the intraplantar formalin test confirmed that both drugs were able to attenuate neurogenic pain (*F*[2,21] = 14.92, *p* < 0.0001) with morphine being slightly more effective (Fig. 4a). The antinociceptive activity of both tested agents was also shown in the second (inflammatory) phase which demonstrated that cebranopadol and morphine significantly (at *p* < 0.01 and *p* < 0.001, respectively) reduced pain responses in formalin-treated animals (*F*[2,21] = 11.81, *p* < 0.001; Fig. 4b).

### Chemotherapy-induced neuropathic pain model

#### Influence on cold allodynia (single-drug administration protocol)

**Fig. 5:**
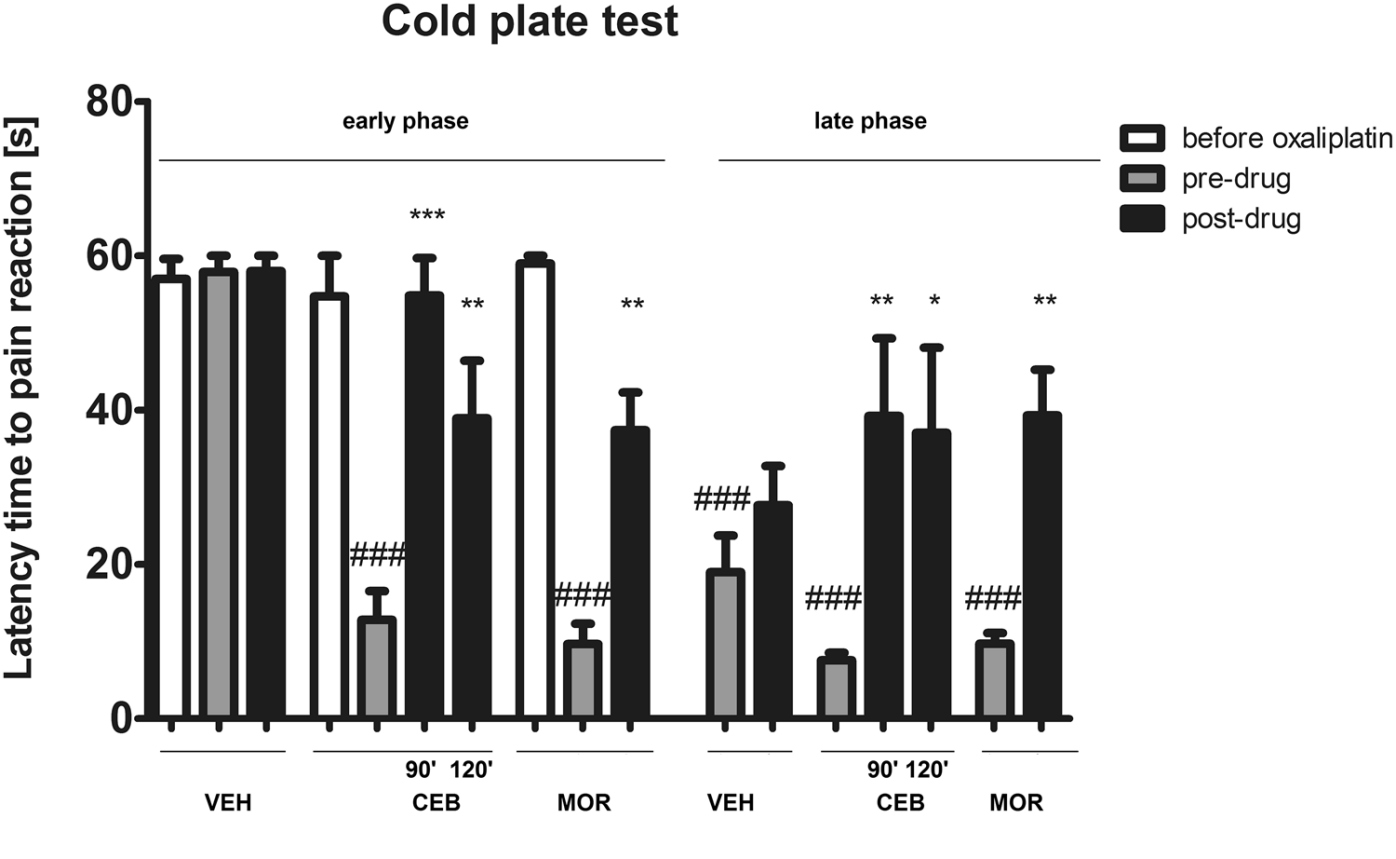
Antiallodynic activity of cebranopadol and morphine in oxaliplatin-induced neuropathic pain model. Cold allodynia was measured using the cold plate test in the early phase (3 h after oxaliplatin administration) and in the late phase (7 days after oxaliplatin administration). Results are shown as latency time to pain reaction (±SEM) in response to thermal stimulation (temperature of 2 °C). Statistical analysis: one-way analysis of variance, followed by Tukey’s post hoc test. Significance vs. latencies of vehicle-treated mice (i.e., mice not treated with oxaliplatin): ^###^ *p* < 0.001, and vs. pre-drug latencies at the respective time-point: *p < 0.05, **p < 0.01, ***p < 0.001

In this experiment, an overall effect of treatment was observed (*F*[16,127] = 15.58, *p* < 0.0001). As shown in Fig. 5, during the early phase cold allodynia, both cebranopadol and morphine demonstrated strong and statistically significant (*p* < 0.01) antiallodynic properties. Their antiallodynic activity was also confirmed during the late phase of cold allodynia—7 days after oxaliplatin injection (significant at *p* < 0.05 and *p* < 0.01 for cebranopadol and morphine, respectively).

#### Influence on cold allodynia (combined treatment protocol: cebranopadol and simvastatin)

Preliminary experiments (data not shown) performed using the cold plate test in mice not treated with oxaliplatin (i.e., non-neuropathic mice) revealed overall effects of single-dose and repeated-dose simvastatin treatments (single-dose simvastatin: *F*[5,32] = 8.366, *p* < 0.0001; repeated-dose simvastatin: *F*[7,46] = 4.209, *p* < 0.01). *Post hoc* analyses revealed that there were significant (*p* < 0.001) differences between the latencies of naïve mice and those of mice treated with a single dose of simvastatin. The latencies of the latter group were significantly reduced as compared to naïve mice. Similar effects were observed in mice repeatedly treated with simvastatin (*p* < 0.01 vs. naïve mice). No significant differences were noted when the latencies of mice treated with single-dose and repeated-dose simvastatin were compared (*F*[5,46] = 0.3205, *p* > 0.05).

**Fig. 6:**
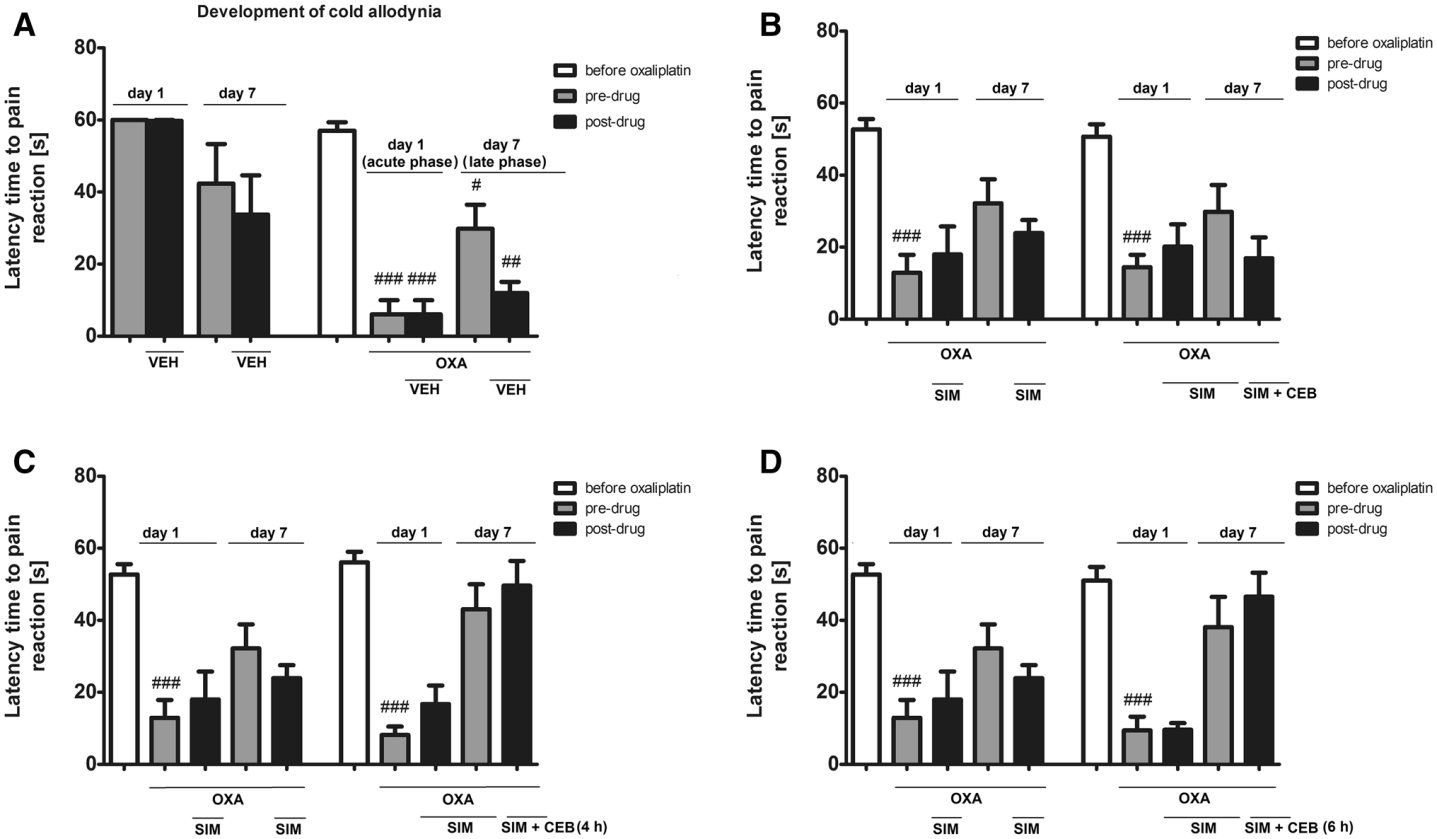
Influence of oxaliplatin (10 mg/kg, i.p.; OXA), simvastatin (100 mg/kg, oral route; SIM) alone, or in combination with subcutaneous cebranopadol (10 mg/kg, s.c.; CEB) on cold sensitivity threshold and cold allodynia measured in the cold plate test in oxaliplatin-induced neuropathic pain model. Effect of oxaliplatin on the development of cold allodynia (**a**). Effect of 7-day administration of oral simvastatin alone (OXA + SIM), or in combination with single-dose cebranopadol added on day 7: simultaneously (**b** OXA + SIM + CEB), 4 h (**c** OXA + SIM + CEB (4 h)), or 6 h (**d** OXA + SIM + CEB (6 h)) after simvastatin. Results are shown as latency time to pain reaction (±SEM) in response to thermal (cold) stimulation (2 °C). In this test, the data were collected at two time-points: on day 1 to measure the latencies to pain reaction during the acute-phase cold allodynia that developed after oxaliplatin injection, and on day 7 to measure the latencies to pain reaction during the late-phase cold allodynia. Day 1: grey bars indicate baseline latencies of oxaliplatin-treated mice measured before drug treatment (i.e., before vehicle or simvastatin administration; defined as ‘pre-drug’) and black bars depict latencies after drug administration (i.e., after vehicle or simvastatin administration; referred to as ‘post-drug’ values). Day 7: grey bars indicate baseline latencies of oxaliplatin-treated mice measured before drug treatment (i.e., before vehicle, simvastatin or cebranopadol administration; defined as ‘pre-drug’) and black bars depict latencies after drug administration (i.e., after vehicle, simvastatin only, or simvastatin and cebranopadol administration; referred to as ‘post-drug’ values). A detailed protocol used is also shown in Fig. 1. Statistical analysis: one-way analysis of variance, followed by Tukey’s post hoc test. Significance vs. latency to pain reaction before oxaliplatin injection: ^##^ *p* < 0.01, ^###^ *p* < 0.001.

The administration of oxaliplatin significantly reduced cold sensitivity threshold in mice (*F*[8,31] = 11.48, *p* < 0.0001). Significantly decreased latency time to pain reaction (*p* < 0.001 vs. values before oxaliplatin administration) was observed 3 h after oxaliplatin injection. This indicated for the development of cold allodynia in oxaliplatin-treated, neuropathic mice (Fig. 6a). Cold allodynia was maintained even for 7 days (significant at *p* < 0.05 vs. values before oxaliplatin administration).

On day 1 in neuropathic (oxaliplatin-treated) mice, simvastatin administration (black bars—Figs. 6b–d: sim + oxa) did not influence cold allodynia significantly, although we observed a slight prolongation of latency time to pain reaction after a single dose of this drug. Noteworthy, comparing pre-drug latencies in the cold plate test (grey bars—Figs. 6b–d: sim + oxa) during the early (on day 1) and the late (on day 7) phases of cold allodynia to the latency of untreated controls (a white bar—Figs. 6b–d: sim + oxa), it can be concluded that the repeated (7-day) administration of simvastatin partially reversed the decrease of pain threshold caused by oxaliplatin and partially restored it to values of mice not treated with oxaliplatin.

In general, the use of cebranopadol as ‘add-on’ therapy with simvastatin in neuropathic, oxaliplatin-treated mice showed no benefits. However, we noted some differences among groups treated with ‘add-on’ cebranopadol used simultaneously (*F*[9,70] = 6.657, *p* < 0.0001; Fig. 6B: sim + ceb), 4 h (*F*[9,65] = 11.37, *p* < 0.0001; Fig. 6C: sim + ceb (4 h)) or 6 h (*F*[9,65] = 9.875, *p* < 0.0001; Fig. 6d: sim + ceb (6 h)) after simvastatin. *Post hoc* analyses showed no significance of these results but interestingly, we observed a trend towards the prolongation of latency time to pain reaction in mice treated with combined simvastatin and cebranopadol, when cebranopadol was administered 4 h (Fig. 6c), or 6 h after simvastatin (Fig. 6d), but not if these two drugs were given simultaneously (Fig. 6b).

### Influence on motor coordination (rotarod test)

In the rotarod test, neither cebranopadol nor morphine influenced animals’ motor coordination at 6, 18, or 24 rpm which means that the drugs tested did not cause motor deficits in mice.

## Discussion

In this study, we implemented mouse models of acute, tonic, and chronic pain to assess antinociceptive properties of cebranopadol, a dually acting nociceptin/orphanin FQ and opioid receptor agonist. Antinociceptive efficacy of cebranopadol was compared to that of morphine used at an equal dose. A summary of results obtained for both drugs in various mouse pain tests is presented in Table 1.

**Table 1:** Comparison of antinociceptive properties of cebranopadol and morphine in mouse models of pain

Pain test	Efficacy: cebranopadol (CEB) vs. morphine (MOR)	Figure
Hot plate test (acute thermal pain)	CEB = MOR^a^	Figure 2
Writhing test (acute inflammatory pain)	CEB = MOR	Figure 3a
Capsaicin test (acute neurogenic pain)	CEB = MOR	Figure 3b
Formalin test (tonic—neurogenic and inflammatory pain)	CEB < MOR	Figure 4a,b
Cold plate test (oxaliplatin-induced neuropathic pain)	CEB ≥ MOR	Figure 5

^a^Data from Gades et al. 2000; Sałat et al. 2012

In the hot plate test, both cebranopadol and morphine demonstrated strong and statistically significant antinociceptive properties. Of note, the activity of cebranopadol was delayed as compared to that previously shown for morphine (90–120 min vs. 30–60 min for cebranopadol and morphine, respectively) (Gades et al. 2000; Sałat et al. 2012). The hot plate assay is a rodent model of acute pain. The paws of mice are very sensitive to heat at temperatures that are not harmful to the skin. The characteristic responses such as jumping, licking of the paws are of central origin and it is thought that drugs with antinociceptive properties in the hot plate test act primarily in the spinal medulla and/or higher central nervous system levels (Vogel and Vogel 1997). In this assay, peripherally acting analgesics are generally not active (Vogel and Vogel 1997). Thus, the results obtained in the present study confirmed the role of central opioidergic system in mediating analgesia caused by cebranopadol (and morphine).

Available literature data indicate that functional NOP and MOP receptors are expressed not only at spinal and supraspinal sites of the ascending and descending pain pathways but also in the periphery. NOP receptors and their ligand—N/OFQ have been found in many peripheral organs (e.g., airways and cardiovascular system) and in the immune system in rodents and humans (Schröder et al. 2014). In a rat model of inflammatory pain (i.e., trinitrobenzene sulfonic acid (TNBS)-induced colonic hyperalgesia), N/OFQ demonstrated antihypersensitive effects after peripheral administration and it was antinociceptive in the capsaicin test in mice (Sakurada et al. 2005). N/OFQ exerted analgesic properties in the tail-flick test in rats (Xu et al. 1996; Tian et al. 1997) and mice (King et al. 1997). Moreover, spinal N/OFQ potentiated analgesia caused by systemic morphine (Tian et al. 1997), while a selective non-peptide NOP receptor agonist, SCH-221510, showed anti-inflammatory and analgesic properties in a mouse model of TNBS-induced inflammatory bowel disease after systemic administration (Sobczak et al. 2013, 2014).

In line with these findings, in our present study, both cebranopadol and morphine attenuated chemogenic, inflammatory acute pain responses induced by acetic acid. Since the writhing test is regarded a rodent model of pain mediated by peripheral mechanisms related to inflammation (Vogel and Vogel 1997), it seems plausible that peripheral NOP and MOP receptors might play a role in the observed activity of both drugs. The involvement of NOP receptors in the pathophysiology of inflammation, arterial hypertension, and cardiac or brain circulatory ischemia has been reported previously (reviewed in Schröder et al. 2014). In the rat model of carrageenan-induced inflammation, i.t. N/OFQ inhibited thermal hyperalgesia (Yamamoto et al. 1997b; Hao et al. 1998), and NOP receptors and their endogenous ligand—N/OFQ modulated neurogenic inflammation and other functions, such as airway tone and caliber (Singh et al. 2016).

The influence of cebranopadol and morphine on peripherally expressed opioid receptors might also explain their activity observed in the capsaicin test. This pain test reflects acute inflammatory pain responses related to neurogenic inflammation. Capsaicin is an exogenous activator of the TRPV1 channels present in sensory neurons, mainly in C-fibers and, to a lesser extent, Aδ. It shows a biphasic effect, i.e., it stimulates TRPV1 located in sensory neurons, resulting in a rapid phase of neurogenic pain with a burning sensation, local vascular and extravascular responses, after which persistent desensitization with concomitant long lasting analgesia appears (reviewed in Sałat et al. 2013b; Marwaha et al. 2016). Previously, it was shown that NOP receptor activation abolished capsaicin-induced contraction of guinea pig airways (Shah et al. 1998; Corboz et al. 2000), reduced capsaicin-induced bronchoconstriction, and increased airway hyper-responsiveness in ovalbumin-sensitized mice (D’Agostino et al. 2010). Of note, in the peripheral nervous system, N/OFQ inhibited neurotransmitter release (Giuliani et al. 2000) and inhibited substance P-mediated nociception in mice (Inoue et al. 1999). Considering an important role of substance P in neurogenic inflammation caused by capsaicin, the antinociceptive activity of cebranopadol in the capsaicin test can be explained in terms of its influence on neurogenic inflammation.

In the formalin test—a tonic pain model, cebranopadol was slightly less active than morphine. This was observed both in the acute (neurogenic) phase, and in the late (inflammatory) phase of this test. Intraplantar formalin induces chemogenic pain that results from neurogenic inflammation, sensory C-fibers activation, as well as sensitization within the spinal cord dorsal horn and the brain (Hunskaar and Hole 1987; Tjølsen et al. 1992; Yashpal and Coderre 1998), but there is also evidence that formalin is a potent stimulator of TRPA1 channels (McNamara et al. 2007; Nassini et al. 2015; Sałat and Filipek 2015). Our present study shows that NOP/MOP receptor activation might also mediate analgesia in the formalin test. It was previously demonstrated that in the formalin test, N/OFQ was antinociceptive after intrathecal administration, whereas it exerted pronociceptive effects and antagonized opioid analgesia when administered intracerebroventricularly (Erb et al. 1997; Yamamoto et al. 1997a; Zhu et al. 1997; Hao and Ogawa 1998; Wang et al. 1999). This bidirectional and site-specific modulation of nociception was also confirmed in the mouse formalin test in which UFP-101, a peptide antagonist selective for NOP receptors, exerted antinociceptive and pronociceptive effects after intracerebroventricular and intrathecal administration, respectively (Rizzi et al. 2006). Antihyperalgesic activity in the formalin test was also observed after systemic administration of selective, non-peptide NOP receptor agonist GRT-TA2210 (Linz et al. 2013).

In animals treated with oxaliplatin, the pain threshold for cold nociception is significantly lower as compared to non-treated mice. This was confirmed in our study in the cold plate test and indicated for the development of cold allodynia in oxaliplatin-treated mice. Recently, it has been shown that tactile allodynia and cold allodynia in rodents treated with oxaliplatin are mediated by TRPA1 stimulation (Nassini et al. 2011; Zhao et al. 2012; Sałat et al. 2013b; Marwaha et al. 2016) and a single dose of oxaliplatin induces acute cold hypersensitivity associated with an enhanced responsiveness of TRPA1 channels (Zhao et al. 2012). The antiallodynic activity of cebranopadol (and morphine) in the oxaliplatin model of neuropathic pain, in particular in the acute phase, together with the results obtained in the formalin test indicate that both drugs tested are able to attenuate TRPA1-mediated pain responses in mice. To the best of our knowledge, this drug has not been evaluated in these pathological conditions previously and our present study confirmed its potential utility for patients suffering from oxaliplatin-induced neuropathic pain. This is a potentially interesting finding as classical opioid drugs have limited efficacy in neuropathic pain conditions and they are regarded the third-line (morphine) or the second-line (tramadol) treatment for neuropathic pain (Finnerup et al. 2015), which is in part due to a diversity of mechanisms underlying the development of neuropathic pain (Torrance et al. 2013).

The expression of NOP receptors is up-regulated in chronic (neuropathic and inflammatory) pain conditions (Briscini et al. 2002; Chen and Sommer 2006; Schröder et al. 2014) and N/OFQ showed antihypersensitive effects in various rodent models of neuropathic pain, including rat CCI model (Yamamoto et al. 1997b; Corradini et al. 2001; Courteix et al. 2004), spinal nerve ligation (SNL) model (Ju et al. 2013), as well as the mouse diabetic neuropathic pain model (Kamei et al. 1999). In addition, some non-peptide NOP receptor agonists (SR14150 and SR16835) displayed NOP receptor-dependent antiallodynic activity in SNL model in mice (Khroyan et al. 2011), whereas in the mouse CCI model, GRT-TA2210 and Ro65-6570 demonstrated strong antiallodynic effects after spinal, supraspinal, and systemic administration (Linz et al. 2013).

Strong anti-inflammatory activity of cebranopadol, as well as the previously reported role of MOP, NOP receptors and N/OFQ in the attenuation of inflammation led us to investigate, if cebranopadol might potentiate/modulate the antiallodynic activity of other drugs with anti-inflammatory properties in chronic pain conditions. For this purpose, we chose the oxaliplatin neuropathic pain model and we used simvastatin as a potential novel treatment option for this pharmacoresistant pain type. Among numerous pathomechanism inflammation has been discovered as one of the key factors underlying neurotoxicity of oxaliplatin (Massicot et al. 2013; Waseem and Parvez 2016). Anti-inflammatory and analgesic properties of simvastatin in neuropathic pain conditions have been widely described in the literature (Miranda et al. 2011; Jaiswal and Sontakke 2012). Moreover, it has been shown that statins have protective effect on ultrastructural alterations induced by cold stress in rats (Bombig et al. 2003). They are also able to attenuate mitochondrial injury induced by cold exposure, inhibit the elevation of blood pressure in cold-treated mice via the downregulation of Bcl-2 pathway (Liang et al. 2017), and they are effective in the attenuation of Raynaud’s phenomenon (Baumhäkel and Böhm 2010). This activity of simvastatin is complex and it involves various pleiotropic effects, such as modulation of endothelial functions and Rho-kinase inhibition, which, in turn, affects TRPM8 activity (Sun et al. 2014). Taken together, these data clearly suggest that statins might modulate body functioning of cold-exposed subjects.

In the cold plate test in oxaliplatin-treated mice compared to non-treated controls, a significant reduction of latency time to pain reaction was observed both 3 h and 7 days after its administration. This indicated the development of cold allodynia in oxaliplatin-treated mice. Cold allodynia in neuropathic mice was not influenced by a single dose of simvastatin administered alone (see results for the early phase), but in contrast to this, a 7-day treatment with this drug partially reversed allodynia induced by cold and the difference between latencies of non-neuropathic mice and oxaliplatin + repeated simvastatin-treated mice was no longer significant. Thus, it might indicate that the repeated administration of simvastatin elevated pain threshold of animals with CIPN. The addition of cebranopadol to simvastatin did not demonstrate additional benefits. Of note, a simultaneous treatment with both drugs reduced latency time to pain reaction. Numerous studies have shown that the hot/cold plate test latencies might decrease with a repeated testing and this decrease may involve learning abilities, differences in animals’ weight, habituation time, and other unknown factors (Czopek et al. 2016). Of note, cebranopadol added to oxaliplatin + repeated simvastatin-treated mice 4 h or 6 h after the last dose of simvastatin (day 7) did not reduce the latency time to pain reaction and a tendency towards the prolongation of this parameter was noted. This is an interesting finding which requires further studies, but it may potentially suggest some pharmacodynamic interaction at the common site of action.

The rotarod test was involved for a proper interpretation of data obtained in pain tests to avoid the possibility of false positive results (Vogel and Vogel 1997). The results of our study showed no motor deficits in mice treated with cebranopadol and this finding is in line with the previous literature data (Bird and Lambert 2015).

To conclude, in this study using mouse models of acute, tonic, and chronic pain, we demonstrated antinociceptive and antiallodynic properties of cebranopadol—a novel, first-in-class agonist at nociceptin/orphanin FQ and opioid receptors. Of note, the antiallodynic activity of cebranopadol in neuropathic pain related to CIPN was shown for the first time, indicating a potential novel treatment option for this type of chronic pain. Apart from this, the results from our present study suggest that NOP receptors might be an important drug target for analgesics used not only in neuropathic pain conditions but also in inflammatory pain. This is of particular relevance in terms of pharmacoresistance of these types of chronic pain to currently available analgesic drugs.
